# A cross-domain framework for emotion and stress detection using WESAD, SCIENTISST-MOVE, and DREAMER datasets

**DOI:** 10.3389/fbioe.2025.1659002

**Published:** 2025-11-25

**Authors:** Ahmad Almadhor, Stephen Ojo, Thomas I. Nathaniel, Kingsley Ukpong, Shtwai Alsubai, Abdullah Al Hejaili

**Affiliations:** 1 Department of Computer Engineering and Networks, College of Computer and Information Sciences, Jouf University, Sakaka, Saudi Arabia; 2 Department of Electrical and Computer Engineering, College of Engineering, Anderson University, Anderson, SC, United States; 3 School of Medicine Greenville, University of South Carolina, Columbia, SC, United States; 4 Department of Electrical Electronics Engineering, Federal University of Technology, Oye Ekiti, Nigeria; 5 College of Computer Engineering and Sciences, Prince Sattam bin Abdulaziz University, AlKharj, Saudi Arabia; 6 Faculty of Computers and Information Technology, Information Technology Department, University of Tabuk, Tabuk, Saudi Arabia

**Keywords:** biosignal classification, mental health monitoring, stress detection, deep learning, transfer learning, emotion recognition, physiological signals, explainable artificial intelligence (XAI)

## Abstract

**Introduction:**

Emotional and stress-related disorders pose a growing threat to global mental health, emphasizing the critical need for accurate, robust, and interpretable emotion recognition systems. Despite advances in affective computing, existing models often lack generalizability across diverse physiological and behavioral datasets, limiting their practical deployment.

**Methods:**

This study presents a dual deep learning-based framework for mental health monitoring and activity monitoring. The first approach introduces a framework for stress classification based on a 1D-CNN trained on the WESAD dataset. This model is then fine-tuned using the ScientISST-MOVE dataset to detect daily life activities based on motion signals, and it is used as transfer learning for a downstream task. An explainable AI technique is used to interpret the model’s predictions, while class imbalance is addressed using focal loss and class weighting. The second approach employs a temporal conformer architecture combining CNN and transformer components to model temporal dependencies in continuous affective ratings of emotional states based on valence, arousal, and dominance (VAD) using the DREAMER dataset. This method incorporates feature engineering techniques and models temporal dependencies in ECG signals.

**Results:**

The deep learning classifier trained on WESAD biosignal data achieved 98% accuracy across three classes, demonstrating highly reliable stress classification. The transfer learning model, evaluated on the ScientISST-MOVE dataset, achieved an overall accuracy of 82% across four activity states, with good precision and recall for high-support classes. However, the explanations produced by Grad-CAM appear uninformative and do not clearly indicate which parts of the signals influence the prediction. The conformer model achieved an R^2^ score of 0.78 and a rounded accuracy of 87.59% across all three dimensions, highlighting its robustness in multi-dimensional emotion prediction.

**Discussion:**

The framework demonstrates strong performance, interpretability, and real-time applicability in personalized affective computing.

## 1 Introduction

Emotions are deeply intertwined with human cognition, perception, and behavior, influencing decision-making, social interactions, and mental wellbeing (Geng et al., 2025). As artificial intelligence becomes increasingly embedded in our daily lives, there is growing interest in developing systems capable of understanding and responding to human emotions (Kukhilava et al., 2025). This has led to the rise of affective computing, an interdisciplinary field combining insights from computer science, psychology, neuroscience, and engineering (Bravo et al., 2025). Among various modalities, Electroencephalography (EEG) and other physiological signals have proven to be especially valuable for emotion recognition due to their direct reflection of internal affective states, which are less prone to cultural bias or conscious control (Shah et al., 2025).

In recent years, deep learning (DL) models have demonstrated remarkable capabilities in decoding complex patterns from biosignals such as EEG, electrodermal activity (EDA), and heart rate variability (HRV) (Gkintoni et al., 2025). Unlike traditional machine learning methods that rely on handcrafted features, deep neural networks automatically learn hierarchical representations, enabling more accurate and robust emotion classification (Prabowo et al., 2023). Architectures such as Convolutional Neural Networks (CNNs), Recurrent Neural Networks (RNNs), and hybrid models have achieved state-of-the-art performance across various benchmarks (Gudikandula et al., 2024). In this study, we focus on emotion classification using deep learning models applied to two widely used and diverse datasets: DREAMER and WESAD. The DREAMER dataset includes EEG signals and is designed for analyzing valence, arousal, and dominance dimensions of emotion, making it suitable for understanding affective brain responses.^1^ In contrast, the WESAD dataset provides multimodal physiological data, including EDA, ECG, and respiration, from wearable sensors, enabling emotion detection in more realistic, everyday settings.^2^ By leveraging both datasets, we aim to explore the effectiveness of deep learning techniques across different signal modalities and emotional representations.

Despite growing interest and success, the field faces challenges such as signal variability, limited dataset sizes, inconsistent preprocessing techniques, and a lack of standardized evaluation protocols (Vafaei and Hosseini, 2025). This work addresses these gaps by systematically applying and comparing deep learning models on both EEG and physiology-based emotion recognition tasks. Our goal is to contribute to the development of more generalizable and interpretable emotion-aware systems that can support applications in mental health monitoring, adaptive human-computer interaction, and real-time affective feedback.

The key contributions of this study are as follows:

- Proposed a dual deep learning-based framework comprising a model for stress classification and transfer learning for activity state recognition and a Temporal Conformer architecture for continuous emotion prediction using physiological signals.
- Developed a pipeline using the WESAD dataset, where a 1D-CNN was trained and then fine-tuned on another ScientISST-MOVE dataset via transfer learning to enhance cross-domain generalization.
- Introduced a temporal conformer architecture utilizing the DREAMER dataset to predict continuous emotional states through valence, arousal, and dominance (VAD) scores, capturing temporal dependencies in affective signals.
- Integrated explainable AI techniques: Grad-CAM, Integrated Gradients, and attention visualization to interpret and validate model decisions, increasing transparency and trustworthiness.
- The proposed unified deep learning framework combining 1D-CNN classifiers with transfer learning and a temporal conformer architecture (CNN + Transformer) was evaluated on the WESAD (stress), ScientISST-MOVE dataset (activity state recognition), and DREAMER (emotion) datasets, achieving accuracies of 98%, 82%, and 87.59%, respectively.

This research paper presents a comprehensive investigation of a hybrid framework for emotion and stress recognition using physiological signal data. The related work Section 2 examines prior work in emotion recognition and physiological signal analysis, highlighting existing deep learning and machine learning methods and their limitations in interpretability. The proposed framework Section 3 outlines the dual proposed approaches, also elaborates on data preprocessing, feature extraction, model architecture, and techniques for handling class imbalance. The results and discussion Section 4 presents a comparative analysis of model performance using standard evaluation metrics, supported by interpretability visualizations from XAI methods such as Grad-CAM and Integrated Gradients. Section 5 demonstrates future work and limitations. Finally, the conclusion Section 6 summarizes the key contributions and outlines future directions to enhance the framework’s real-world applicability in affective computing and mental health monitoring.

## 2 Related work

This section highlights recent studies that have applied advanced machine learning (ML), deep learning (DL), and Explainable AI (XAI) techniques to emotion recognition using physiological signals. These efforts focus on enhancing classification performance, interpretability, and real-time monitoring of emotional states.

Electrodermal Activity (EDA), which reflects the skin’s electrical properties in response to psychological arousal, has emerged as a central biosignal for stress detection. In Medikonda and Rai (2024), six time-domain features were extracted from EDA signals, and K-means clustering was used to categorize the data into three stress states: low, moderate, and high. Several classifiers were then applied to evaluate the accuracy of these labeled stress levels, with Decision Tree achieving the best precision, and Random Forest and Naive Bayes reaching up to 93% accuracy, followed by Support Vector Machine at 86%. Recent studies have shifted towards personalized deep learning approaches for emotion classification using physiological signals. Authors in Li et al. (2024) utilized the WESAD dataset and demonstrated that a personalized model significantly outperforms generalized models in classifying three emotional states: neutral, stress, and amusement. The customized model achieved 95.06% accuracy and an F1-score of 91.71%, while participant-inclusive and participant-exclusive generalized models lagged with accuracies around 67% and lower F1-scores. These results underscore the effectiveness of individual-specific model tuning in capturing subtle physiological variations related to emotional states.

In addition to model performance, data privacy is a growing concern in biosignal-based stress detection. To mitigate these concerns (Tanwar et al., 2022), proposed a federated learning-based framework that enables decentralized model training on edge devices without requiring the transmission of user data to a central server. Using a deep neural network and the WESAD dataset, the study achieved an accuracy of 89.69%, successfully balancing model performance with data confidentiality. Experiments across multiple communication rounds with five simulated clients revealed the robustness and privacy-preserving nature of this distributed learning strategy. Meanwhile (Warrier et al., 2024), explored the feasibility of stress detection using low-resolution EDA signals from consumer-grade wearables. A statistical analysis comparing user-dependent and user-independent models indicated that user-dependent approaches are statistically more accurate. Interestingly, using low-resolution EDA signals did not significantly degrade performance compared to high-resolution data, highlighting the potential of affordable, scalable stress-monitoring systems for everyday use.

Another promising direction involves hybrid deep learning architectures. In Ninh et al. (2021), a CNN-LSTM model was proposed for classifying stress into three categories: baseline, stress, and amusement. The approach integrated multiple physiological signals, including ECG, EMG, respiration, temperature, and EDA, demonstrating an improved classification accuracy of 90.20%. The model’s ability to learn spatial and temporal features from multimodal biosignals suggests its applicability across diverse real-world scenarios, including office environments, driving conditions, and personal health monitoring. Authors in Bansal et al. (2025) employed the WESAD dataset, comprising data from 15 subjects, to classify stress levels based on physiological signals and accompanying questionnaires. A novel method, StressNet-GAF, was proposed that utilizes Gramian Angular Field (GAF) image generation and the pre-trained VGG-16 model via transfer learning. This approach achieved 91% accuracy across three stress categories. Future directions include exploring data augmentation techniques to improve generalization and reduce overfitting during model training.

Recent advancements in emotion recognition have highlighted the potential of multimodal biosignal data, particularly EEG and ECG, for accurately detecting human emotional states. Authors in Ye et al. (2024) conducted a comparative analysis of various machine learning classifiers using the DREAMER dataset. Emotions were classified through different schemes: binary, PNN (positive-neutral-negative), 2D valence-arousal, and 3D VAD (valence-arousal-dominance). The ensemble of SVM and Random Forest achieved approximately 80% accuracy for binary emotions. At the same time, Multinomial Logistic Regression and Random Forest consistently maintained 80%–90% accuracy across both binary and non-binary settings, outperforming the dataset’s original benchmarks. Multimodal fusion models, particularly deep canonical correlation analysis (DCCA) and bimodal deep autoencoder (BDAE), have shown superior performance in integrating heterogeneous physiological features. Authors in Liu et al. (2021) extended DCCA with weighted and attention-based fusion strategies and evaluated these models on five datasets, including SEED, SEED-IV, DEAP, SEED-V, and DREAMER. DCCA consistently outperformed alternatives, achieving recognition rates of up to 94.6% (SEED) and 90.7% (DREAMER), while demonstrating resilience to noise in EEG channels, highlighting its robustness and discriminative feature-learning capabilities.

The author in Abdelfattah et al. (2025) utilized the WESAD dataset, comprising physiological signals from 15 subjects, including ACC, ECG, BVP, TEMP, RESP, EMG, and EDA, to classify four states: baseline, stress, amusement, and meditation. Seven traditional machine learning models (e.g., Random Forest, XGBoost) and three deep learning models (DNN, CNN, RNN) were evaluated in two experimental phases. Results showed that RNN achieved a 93% F1 score when tested across subjects, while Random Forest, Extra Trees, and XGB classifiers reached 99% F1 scores in subject-specific evaluations. Chest sensor data yielded better performance in subject-dependent setups, whereas wrist data performed better in cross-subject scenarios. Authors in Topic and Russo (2021) introduced a novel emotion recognition approach using topographic (TOPO-FM) and holographic (HOLO-FM) feature maps of EEG signals. Deep learning was employed for feature extraction and subsequent fusion, followed by classification. Experiments on DEAP, SEED, DREAMER, and AMIGOS datasets demonstrated that TOPO-FM and HOLO-FM representations enhanced emotion classification in the two-dimensional emotional space, surpassing existing EEG-based recognition models. A deep forest model for EEG-based emotion recognition was proposed in Cheng et al. (2020), emphasizing spatial-temporal dynamics through 2D frame sequences and baseline signal removal. Unlike traditional methods, this approach required no manual feature engineering and proved highly effective. On the DREAMER dataset, it achieved impressive accuracies of 89.03% (valence), 90.41% (arousal), and 89.89% (dominance), outperforming state-of-the-art techniques and demonstrating adaptability to EEG signal variability.

Authors in Singh and Singh (2023) explored subject-dependent and subject-independent evaluation perspectives using a DCNN-based feature extractor combined with multiple classifiers across three emotion labeling schemes, binary, quad, and octal, on DEAP and DREAMER datasets. Subject-independent models exhibited more robust performance, particularly with the DCNN+NN and DCNN+SVM pipelines, suggesting that arousal and dominance are more influential than valence in emotion recognition, contrary to prior findings. Authors in Gokalp et al. (2024) introduced hyperparameter-tuned deep learning architectures, specifically the Special Convolutional Model (SCM) and 2D LSTM, for enhanced EEG-based emotion recognition. On the DREAMER dataset, optimization boosted accuracy from 28% (using WEKA) to 44%, while SCM achieved 64.35% on SEED-IV. These findings demonstrate the crucial role of hyperparameter tuning, particularly with optimizers such as RMSprop, in improving deep learning performance for emotion detection.

Table 1 summarizes state-of-the-art approaches reported in the literature, outlining the datasets used, model architectures, and key findings relevant to affective computing and mental health analysis.

**TABLE 1:** Summary of the literature review.

References	Dataset	Model/technique	Key findings
Bansal et al. (2025)	WESAD (15 subjects)	StressNet-GAF (GAF image generation + VGG-16 with transfer learning)	Achieved 91% accuracy in classifying stress into three categories. The proposed model is effective for early stress detection. Future work includes applying data augmentation to reduce overfitting.
Abdelfattah et al. (2025)	WESAD (15 subjects)	Traditional ML (RF, XGBoost, etc.) and DL models (DNN, CNN, RNN)	RNN achieved 93% F1 score in cross-subject classification. Chest data performed better in subject-dependent setups; wrist data in cross-subject scenarios.
Medikonda and Rai (2024)	Custom EDA Dataset	K-means clustering + DT, RF, NB, SVM classifiers	Decision Tree gave best precision; RF and NB achieved up to 93% accuracy in stress level classification.
Li et al. (2024)	WESAD	Personalized ML model vs. generalized models	Personalized model significantly outperformed generalized ones with 95.06% accuracy and 91.71% F1-score.
Tanwar et al. (2022)	WESAD	Federated learning with CNN on edge devices	Achieved 89.69% accuracy; ensured privacy by training without central data transmission.
Warrier et al. (2024)	Consumer-grade EDA (Low-res)	Statistical analysis on user-dependent and independent models	User-dependent models showed higher accuracy; low-res EDA signals performed comparably to high-res data.
Ninh et al. (2021)	Not specified	CNN-LSTM using multiple signals (ECG, EMG, Respiration, Temp, EDA)	Classified stress into three categories with 90.20% accuracy, leveraging spatial and temporal features.
Ye et al. (2024)	DREAMER	Ensemble (SVM + RF), MLR, RF	Ensemble and SVM achieved 80% for binary emotions; MLR and RF achieved 80%–90% for all VAD-based schemes.
Liu et al. (2021)	SEED, SEED-IV, DEAP, SEED-V, DREAMER	Deep Canonical Correlation Analysis (DCCA), BDAE, attention-based fusion	DCCA achieved up to 94.6% (SEED) and 90.7% (DREAMER); robust to noise; best multimodal fusion model.
Topic and Russo (2021)	DEAP, SEED, DREAMER, AMIGOS	TOPO-FM and HOLO-FM + deep learning feature fusion	Proposed feature maps improved emotion recognition in 2D space across multiple datasets.
Cheng et al. (2020)	DEAP, DREAMER	Deep Forest with 2D frame sequences and baseline removal	No feature extraction required; achieved 90.41% (arousal), 89.89% (dominance) on DREAMER.
Singh and Singh (2023)	DEAP, DREAMER	DCNN + multiple classifiers (NN, SVM, etc.)	Subject-independent models were more robust; DCNN+SVM performed well for multi-class emotion labeling.
Gokalp et al. (2024)	SEED-IV, DREAMER	SCM and 2D LSTM + RMSprop optimization	SCM achieved 64.35% (SEED-IV); DREAMER accuracy improved from 28% to 44% post optimization.

## 3 Proposed framework

**FIGURE 1:**
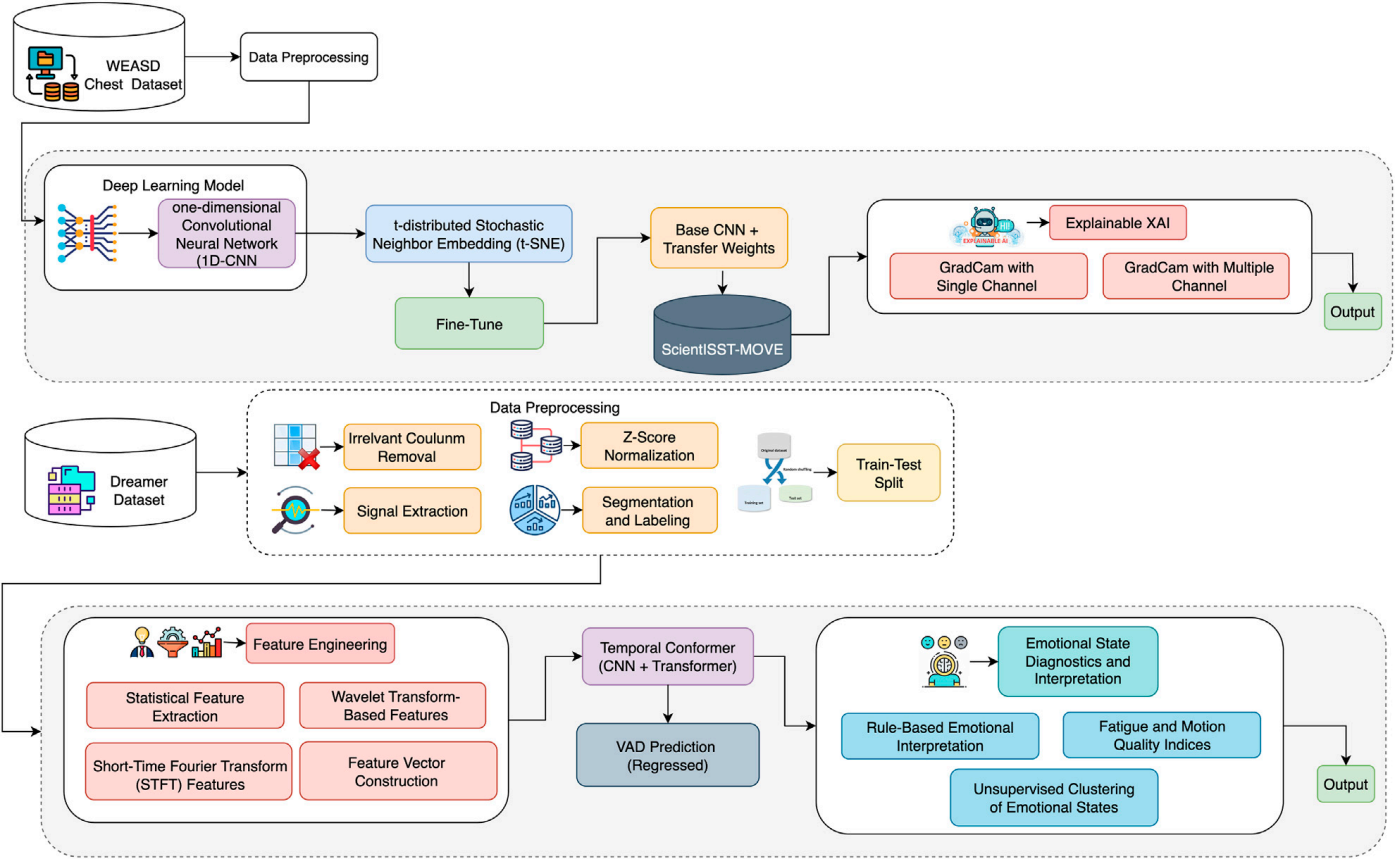
Proposed framework for stress and emotion detection.

This section presents a proposed hybrid framework for stress classification and transfer learning for activity-state recognition, as well as a Temporal Conformer architecture for continuous emotion prediction using physiological signals. The framework is divided into two distinct yet complementary approaches designed to enhance both performance and interpretability. The first approach is a pipeline based on transfer learning, where a 1D-CNN model is pretrained on the WESAD dataset and fine-tuned using a ScientISST-MOVE dataset. This path incorporates advanced signal preprocessing, feature engineering, and attribution-based XAI techniques such as Grad-CAM and Integrated Gradients. The second approach uses a Temporal Conformer model that combines CNN and Transformer modules to predict continuous emotional dimensions, such as valence, arousal, and dominance (VAD), on the DREAMER dataset. Together, these approaches form a comprehensive framework that addresses cross-domain adaptability, class imbalance, and explainability. An overview of the complete architecture is illustrated in Figure 1.

### 3.1 WESAD dataset and preprocessing

The Wearable Stress and Affect Detection (WESAD) is a publicly available dataset designed to support research in emotion recognition and stress detection using physiological signals (Schmidt et al., 2018). The data were collected from 15 participants who underwent a series of controlled experiments designed to induce different emotional states, including Class 0-baseline (neutral), Class 1- stress, and Class 2-amusement. The chest sensor recorded signals from eight channels: electrocardiogram (ECG), electrodermal activity (EDA), electromyogram (EMG), respiration, skin temperature, and three-axis acceleration (Acc_X, Acc_Y, Acc_Z), all sampled at 700 Hz. These signals provide rich temporal data suitable for time-series analysis and for developing machine learning models for affective computing. Each data file includes multiple columns corresponding to these sensor signals, along with associated labels indicating the emotional state during recording. Overall, the dataset comprises several million rows, making it highly valuable for training deep learning models and evaluating real-time stress recognition systems.

Data preprocessing is a crucial step in machine learning, transforming raw data into a clean, usable format for model training and development. In this study, several preprocessing techniques were applied sequentially, including the removal of irrelevant columns, label extraction, Z-score normalization, and the creation of training, validation, and testing sets.

Irrelevant Column Removal: In many datasets, not all columns are useful for model training. Some may be identifiers or contain metadata that do not help in classification or prediction tasks. Removing these helps reduce noise and improve model performance. We dropped non-feature columns such as ‘subject id,’ ‘condition,’ ‘SSSQ class,’ ‘SSSQ Label,’ and ‘condition label’ to isolate the relevant physiological signals and HRV features for classification.

Z-score Normalization: Z-score normalization, also known as standardization, is a technique used to scale features so that they have a mean of 0 and a standard deviation of 1 (Fei et al., 2021). This helps ensure that all features contribute equally to the learning process, particularly for models that are sensitive to feature scaling. The transformation is done using Equation 1.

$$z=\frac{\left(X-\mu \right)}{\sigma }$$ (1)

where X is the original feature value, μ is the mean of the feature, and σ is the standard deviation. In this study, we applied the standard scaler scaling method to standardize the feature values before feeding them into the model.

Train-Validation-Test Split: Splitting the dataset into training, validation, and testing subsets is essential for model development and evaluation. The training set is used for learning, the validation set for tuning hyperparameters, and the testing set for evaluating the final model’s performance. In this study, we first split the data into 70% training (94955, 62) and 30% temporary data. Then, we split the temporary data equally into 15% validation (20347, 62) and 15% test sets (20348, 62), using stratify = labels to maintain label distribution across splits.

#### 3.1.1 1D-CNN architecture

To perform multi-class classification on the given dataset, we implemented a deep learning model based on a one-dimensional Convolutional Neural Network (1D-CNN). The model was specifically designed to handle time-series or sequential data with an imbalanced class distribution. Initially, the input features were reshaped into a three-dimensional format (samples, timesteps, 1) to meet the requirements of 1D-CNN, enabling convolutional filters to extract temporal patterns.

**FIGURE 2:**
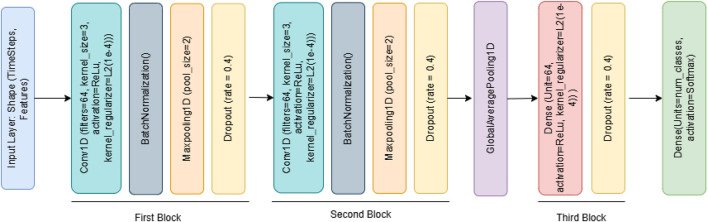
1D-CNN architecture.

Model Architecture: The model architecture comprises two convolutional blocks: the first includes a Conv1D layer with 64 filters (kernel size 3, ReLU activation, L2 regularization), followed by batch normalization, max pooling, and dropout (0.4); the second block similarly uses 128 filters with identical supporting layers (Figure 2). A GlobalAveragePooling1D layer follows, leading to a dense layer with 64 units and a final softmax layer for class probability outputs. The model was compiled using the Adam optimizer (learning rate 0.001) and trained with the sparse_categorical_crossentropy loss function. Training employed early stopping and learning rate reduction strategies based on validation loss trends, with a maximum of 50 epochs and a batch size of 128. To address class imbalance in the dataset, class weights were computed and passed during training. This helps ensure minority classes are not overlooked during the optimization process. The developed 1D-CNN model effectively learned complex representations from the WESAD-Chest dataset. Its trained weights were saved for transfer learning, enabling knowledge reuse across tasks involving multimodal biosignal data.

Algorithm 1 outlines the construction and training process of a 1D Convolutional Neural Network (1D-CNN) model designed for stress classification.

Algorithm 1 outlines the construction and training process of a 1D Convolutional Neural Network (1D-CNN) model designed for stress classification.

Algorithm 1. 1D-CNN Deep Learning Model for Stress Classification

1:  **Input:** Preprocessed signal $X\in R_{n}\times t\times c$

2:  **Output:** Class prediction $\hat{y}$

3:  **Initialize:** CNN model with $L_{2}-regularization$

4:  Add Conv1D (64 filters, ReLU, kernel size = 3)
5:  BatchNorm, MaxPooling1D, Dropout (0.4)
6:  Add Conv1D (128 filters, ReLU, kernel size = 3)
7:  BatchNorm, MaxPooling1D, Dropout (0.4)
8:  GlobalAveragePooling1D
9:  Dense layer (64 units, ReLU), Dropout (0.4)
10:  Dense Softmax layer (number of classes)
11:  **Train:** Use Adam optimizer, sparse categorical crossentropy
12:  Apply EarlyStopping and ReduceLROnPlateau callbacks
13:  Use class weights to mitigate imbalance
14:  **Predict:** $\hat{y}=argmax\left(Softmax\left(Model\left(X\right)\right)\right)$

### 3.2 ScientISST-MOVE dataset and preprocessing

The ScientISST-MOVE dataset consists of multimodal biosignals (e.g., ECG, EMG, RESP, GSR) recorded from wearable sensors using the ScientISST Move platform for activity recognition. These signals were stored in European Data Format (EDF) and segmented into fixed-length windows of 5 s with a stride of 2 s for analysis. Each segment was standardized using z-score normalization and labeled by subject identity for model pretraining. Due to institutional privacy constraints, the dataset is not publicly accessible; however, it conforms to standardized biosignal acquisition protocols. In the SCIENTISST-MOVE dataset, this dataset has 14 activity classes, but we utilized 4 activity classes (run, walk_after, baseline, and walk_before_downstairs) because other classes have very low samples.

#### 3.2.1 Fine-tuned transfer learning

Transfer learning is a powerful deep learning technique that enables the adaptation of a pre-trained model from one domain to a related task, facilitating faster training and improved performance, particularly when working with limited data. In the context of biosignal analysis, it allows the reuse of learned representations from physiological data such as that collected via wearable sensors. In this study, a 1D Convolutional Neural Network (1D-CNN), initially trained on the WESAD-Chest dataset, was leveraged for transfer learning by applying its pretrained weights to a new classification task involving the Scientist Move Annotated Wearable Multimodal Biosignals dataset (Saraiva et al., 2024).

Model Architecture: The architecture begins with an input layer followed by a convolutional block consisting of a Conv1D layer with 64 filters (kernel size 3), ReLU activation, L2 regularization, batch normalization, max pooling, and dropout to reduce overfitting. A second convolutional block follows, expanding to 128 filters with similar processing layers. Feature maps are then condensed using a GlobalAveragePooling1D layer. This is followed by a fully connected dense layer with 64 neurons, ReLU activation, and dropout, culminating in a final softmax output layer for multi-class classification. The model was initialized with pre-trained weights to transfer previously learned temporal features and fine-tuned using domain-specific biosignal data. It was compiled using the Adam optimizer with a learning rate of 0.0001 and trained with class weighting, early stopping, and learning rate scheduling to ensure robust and efficient convergence.

### 3.2.2 t-distributed stochastic neighbor embedding (t-SNE)

**FIGURE 3:**
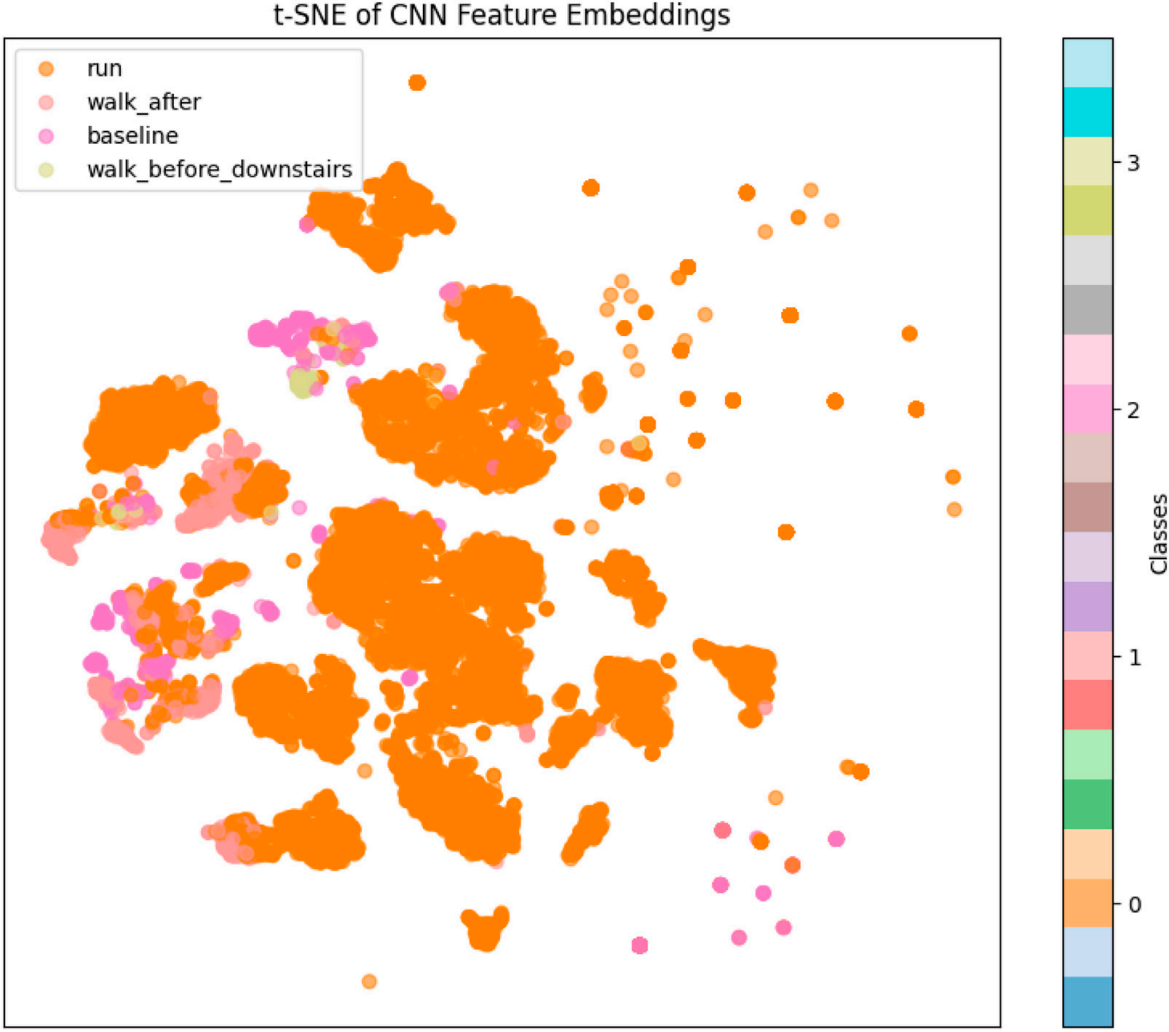
t-SNE of CNN feature embedding.

To gain insights into the internal feature representations learned by the transfer learning model, a t-distributed Stochastic Neighbor Embedding (t-SNE) visualization was employed. This technique was applied to the high-dimensional feature vectors extracted from the test dataset using the trained convolutional neural network (CNN). Specifically, the output of the GlobalAveragePooling1D layer, representing the condensed, abstracted features, was used as input to the t-SNE algorithm. This layer captures the essential patterns and class-specific attributes that the model learns during training, making it ideal for evaluating feature separability. t-SNE is a powerful nonlinear dimensionality reduction technique that projects high-dimensional data into a two-dimensional space while preserving local similarities and relationships among data points. In this study, the t-SNE algorithm was configured with a perplexity of 30 and applied to the extracted features to generate a two-dimensional representation of the test data. Figure 3 shows the resulting 2D feature embeddings, where each point corresponds to a test sample and is color-coded by its ground-truth class label. This visualization provides qualitative evidence regarding the discriminative capability of the learned features. Well-separated, tightly clustered groups indicate that the model has effectively learned class-specific representations, whereas overlapping or dispersed clusters may suggest ambiguity or class confusion.

Algorithm 2 describes the proposed framework developed for Activity Detection using biosignal data. The process begins with preprocessing raw biosignals (in CSV or EDF format), where Z-score normalization and signal filtering (e.g., Butterworth and Notch filters) are applied to enhance data quality. In the second step, a 1D-CNN model is pretrained on HRV (Heart Rate Variability) features from the WESAD dataset, and the learned weights are stored. The third step involves transfer learning, where these pretrained weights are loaded into a new model that is fine-tuned on a different target dataset (ScientISST-MOVE), incorporating class-balancing techniques to address any data imbalance. The fourth step includes model evaluation using standard classification metrics such as accuracy and F1-score. Finally, the framework integrates explainable AI techniques, such as Grad-CAM and Integrated Gradients, to provide interpretability for the model’s predictions, ensuring transparency and trust in the stress classification outcomes.

Algorithm 2. Proposed Framework for Biosignal-Based Activity Detection

1:  **Input:** Raw data (CSV/EDF)
2:  **Output:** Activity Detection with explainability
3:  **Step 1: Preprocessing**
4:  Normalize signals using z-score
5:  Apply filtering (Butterworth, Notch)
6:  **Step 2: Pretraining (WESAD)**
7:  Train 1D-CNN on HRV features
8:  Save weights for reuse
9:  **Step 3: Transfer Learning**
10:  Load weights into new model
11:  Fine-tune on the target dataset with class balancing
12:  **Step 4: Prediction & Evaluation**
13:  Evaluate on test set using Accuracy, F1-score
14:  **Step 5: Explainability**
15:  Apply Grad-CAM and Integrated Gradients for interpretability

#### 3.2.3 Explainable AI (XAI)

XAI refers to a suite of techniques designed to make the decision-making processes of complex machine learning models more transparent and interpretable. In the context of deep learning, XAI methods help reveal which parts of the input data most influenced the model’s predictions, thereby increasing trust, accountability, and understanding in critical domains such as healthcare and neuroscience. In this study, we employed Gradient-weighted Class Activation Mapping (Grad-CAM) to visualize the internal decision process of our models. Specifically, we implemented both single- and multi-channel Grad-CAM visualizations to highlight the regions of the biosignal data that contributed most to the predicted stress or emotional states. These visualizations aid in understanding not only model behavior but also physiological relevance, enhancing model interpretability for clinical and research applications.

### 3.3 DREAMER dataset and preprocessing

The DREAMER dataset is an open-access multimodal dataset designed for emotion recognition research (Ahangaran et al., 2024). It includes electroencephalogram (EEG) and peripheral physiological signals, such as ECG and EMG, recorded from 23 participants (14 male, 9 female) while watching 18 selected video stimuli designed to elicit a range of emotional states. After each video, participants self-reported their valence, arousal, and dominance levels using the Self-Assessment Manikin (SAM) model on a 1–5 scale. A rule-based diagnostic interpretation was applied to the normalized regression outputs, mapping combinations of arousal, valence, and dominance to six interpretable emotional states: Happy/Excited, Fatigued, Stressed, Relaxed, Passive, and Neutral. The dataset supports emotion classification and regression tasks, allowing researchers to explore the relationship between physiological signals and emotional states. It is often used to train and evaluate machine learning and deep learning models for predicting affective states. Preprocessing physiological signals, particularly ECG data, is essential to ensure that the input fed into machine learning models is clean, structured, and informative. In this study, we employed a systematic preprocessing pipeline on the DREAMER dataset, structured into two key stages as follows:

#### 3.3.1 Signal extraction and standardization

The first phase focuses on extracting and preparing the raw ECG signals for analysis.

Extracting ECG Signals: The DREAMER dataset is stored in MATLAB.mat format, which contains structured arrays with nested fields. We began navigating its hierarchical structure to extract the ECG signal from each subject’s trial. Specifically, we accessed the Data field and retrieved the ECG signal from the stimuli subfield. Due to nested object arrays, we implemented an iterative unwrapping process to access raw numerical values. These signals were then converted to NumPy arrays and reshaped into a one-dimensional format for further processing.

Label Extraction: Each trial in the DREAMER dataset includes self-assessed emotional ratings for valence (pleasure), arousal (activation), and dominance (control). These three continuous values were extracted per trial and stored as label vectors, serving as the target outputs.

Z-Score Normalization: To ensure consistency and reduce the influence of individual signal magnitude variations, we applied Z-score normalization to each ECG signal using scikit-learn’s StandardScaler. This transformation centers each signal around a mean of zero and scales it to have a standard deviation of 1. Normalization also improves model convergence and ensures uniformity across all subjects and trials.

#### 3.3.2 Segmentation, labeling, and aggregation

The second phase involves transforming the continuous ECG signals into a structured dataset suitable for machine learning.

Signal Segmentation: The normalized ECG signals were divided into fixed-length windows of 5 s (640 samples at 128 Hz) with 50% overlap, yielding a step size of 2.5 s. This segmentation captures meaningful temporal patterns while increasing the amount of training data through overlapping samples.

Label Assignment to Segments: Each segment inherited the emotional labels (valence, arousal, dominance) of its parent trial. This ensured all windows within a trial were consistently aligned with the correct emotional state, enabling supervised learning on segment-level data.

Final Aggregation: After segmenting and labeling all trials across subjects, the data were compiled into two final NumPy arrays: X for the input features (ECG segments) and y for the corresponding emotional labels. These arrays formed the final dataset used for training and evaluating emotion recognition models.

#### 3.3.3 Feature engineering

Feature engineering is the process of transforming raw data into informative, meaningful features that enhance the predictive performance of machine learning models. In this study, we applied a combination of statistical, time-frequency, and wavelet-based techniques to extract discriminative features from segmented ECG signals derived from the DREAMER dataset.

Statistical Feature Extraction: Each ECG segment was analyzed to extract a set of handcrafted statistical features. These included central tendency (mean, median), dispersion (standard deviation, interquartile range), shape (skewness, kurtosis), and range-based metrics (minimum, maximum). Additionally, we computed the segment’s root mean square (RMS) to capture signal energy and Shannon entropy from a normalized histogram to quantify the signal’s complexity or disorder. These features help describe the overall distribution and variability within the ECG waveform.

Wavelet Transform-Based Features: To capture both the frequency and time-localized characteristics of the ECG signals, we applied the Discrete Wavelet Transform (DWT) using the Daubechies 4 (db4) wavelet up to level 3. From each wavelet sub-band (approximation and detail coefficients), we extracted the mean, standard deviation, and RMS energy. These features are handy for identifying transient changes and non-stationary patterns in ECG signals, which are often associated with emotional states.

Short-Time Fourier Transform (STFT) Features: To further analyze frequency-domain characteristics over short time windows, we applied the Short-Time Fourier Transform (STFT) with a segment size of 64 samples. From the STFT magnitude spectrum, we computed the mean power across the first ten frequency bands. This set of features captures the temporal and spectral power distributions of ECG signals, enabling better modeling of dynamic emotional responses.

Feature Vector Construction: All extracted features from the three domains, statistical, wavelet, and STFT, were concatenated to form a unified feature vector for each ECG segment. The resulting feature matrix, X_features, has a shape of (7291, 33), indicating that a total of 7,291 ECG segments were generated across all subjects and trials, with each segment represented by 33 extracted features. These features include 11 statistical features, 12 wavelet-based features, and 10 STFT-derived frequency domain features. The corresponding label matrix, y, has a shape of (7291, 3, 18, 1), where 3 denotes the emotional dimensions (arousal, valence, dominance), and the additional dimensions pertain to subject-wise and trial-wise structural organization. This transformed dataset serves as the input to the subsequent classification models.

#### 3.3.4 Temporal conformer model

A hybrid deep learning architecture, Temporal Conformer, was developed by integrating 1D convolutional layers with Transformer-based self-attention mechanisms to capture both local feature patterns and long-range temporal dependencies in time-series data. The model is specifically designed to enhance temporal modeling capabilities in multivariate biosignal prediction tasks. The model’s input consisted of 33-dimensional feature vectors, which were first processed by a series of one-dimensional convolutional layers. These convolutional layers act as local feature extractors, capturing spatially correlated patterns across the input feature space. The convolutional stack comprises two sequential layers: the first applies 32 filters, followed by a ReLU activation and dropout for regularization. In contrast, the second layer employs 64 filters with the same activation and dropout configurations as the first layer. The convolutional module’s output is then flattened and projected into a higher-dimensional representation using a fully connected layer, creating a richer embedding space suitable for downstream attention mechanisms.

After the convolutional layers, the model reshapes the output and passes it through a Transformer Encoder. This part of the model features multiple layers that utilize self-attention to focus on key aspects of the input and enhance understanding of relationships over time. A custom Transformer was used to extract attention weights, which help understand what the model is focusing on. Each layer also includes shortcuts (residual connections), normalization, and dropout to stabilize the model and prevent overfitting. The Transformer output is normalized again and passed through a dropout layer before being fed to the final fully connected layer, which produces the model’s three output values. For training, we used the Smooth L1 loss (also known as Huber loss), which is effective at handling outliers. The Adam optimizer was used in conjunction with a scheduler that reduced the learning rate when the validation performance stopped improving.

#### 3.3.5 Emotional state diagnostics and interpretation

Following the prediction of emotional dimensions (arousal, valence, and dominance) using the Temporal Conformer model, a post-processing stage was conducted to interpret these continuous values into more descriptive emotional states, as represented in Table 5.

Rule-Based Emotional Interpretation: A refined set of diagnostic rules was developed to map predicted VAD values to interpretable emotional states, including “Happy/Excited,” “Relaxed,” “Fatigued,” “Stressed,” “Passive,” and “Neutral.” These rules were crafted based on thresholds applied to the arousal and valence dimensions, with dominance considered in cases indicating passivity. For example, high arousal and high valence suggested a happy or excited state, while low arousal and low valence indicated fatigue. This rule-based classification provides intuitive labels that enhance the interpretability of the model’s outputs, especially in affective computing applications.

Fatigue and Motion Quality Indices: To further extract meaningful behavioral insights, two auxiliary metrics were computed. The Fatigue Index is defined as the inverse of the normalized arousal score. This index serves as an estimate of user fatigue, with higher values indicating greater fatigue. On the other hand, the Motion Quality Score is calculated as the product of valence and dominance, providing a measure of how positively and assertively a user interacts or engages with a task. Higher motion quality values suggest a more engaged and confident state, while lower values may indicate reduced interaction quality or passive behavior. These indices provide scalar representations that can be tracked over time or used for downstream decision-making tasks, such as alert systems or adaptive feedback loops in interactive systems.

Unsupervised Clustering of Emotional States: To complement rule-based interpretations, an unsupervised clustering method (K-Means) was applied to the normalized VAD features to group samples into distinct emotional categories based on inherent distribution patterns. Four clusters were chosen empirically, though this parameter can be tuned for specific applications.

Algorithm 3 outlines the proposed Temporal Conformer framework developed for continuous emotion recognition based on Valence, Arousal, and Dominance (VAD) dimensions using raw physiological signals from the DREAMER dataset. The process begins with signal preprocessing, where EEG, ECG, and GSR signals are normalized using z-score and segmented into fixed-length windows aligned with corresponding emotional labels. In the modeling phase, a hybrid architecture is constructed by integrating CNN layers for local feature extraction and Transformer layers for capturing temporal dependencies, forming the Temporal Conformer model. This model is trained to regress continuous VAD values from the sequential signal data. To profile emotional states more comprehensively, unsupervised clustering is applied in the predicted VAD space, followed by the integration of rule-based emotional labeling that incorporates fatigue and motion causality. The model’s performance is evaluated using root mean square error (RMSE) and Pearson’s correlation coefficient, ensuring accurate VAD prediction.

## 4 Result analysis and discussion

This section presents a detailed evaluation of the proposed dual-approach framework for emotion and stress recognition using physiological signals. The performance of deep learning, transfer learning, and the temporal conformer was assessed using a variety of classification and regression metrics, including accuracy, precision, recall, F1-score, confusion matrices, and RMSE, depending on the task. This comprehensive evaluation supports a robust comparison of model performance, interpretability, and generalization across datasets and emotional state categories.

Algorithm 3. Proposed Temporal Conformer Framework for VAD-Based Emotion Recognition

1:  **Input:** Raw physiological signals from DREAMER dataset
2:  **Output:** Continuous emotional prediction (Valence, Arousal, Dominance) with interpretability
3:  **Step 1: Signal Preprocessing**
4:  Normalize EEG, ECG, and GSR signals using z-score
5:  Segment signals into fixed-length windows with labels
6:  **Step 2: Temporal Conformer Modeling**
7:  Construct Temporal Conformer with CNN + Transformer layers
8:  Train model to regress VAD values from signal sequences
9:  **Step 3: Emotional State Profiling**
10:  Apply unsupervised clustering on predicted VAD space
11:  Integrate rule-based emotional labeling and fatigue-motion causality
12:  **Step 4: Evaluation**
13:  Use RMSE and Pearson correlation for VAD prediction assessment

### 4.1 Results of deep learning model (WESAD dataset)

Table 2 presents the classification report for the deep learning model, demonstrating strong performance across all three classes using key evaluation metrics: precision, recall, F1-score, and support. For class 0, the model achieved a perfect precision of 1.00, a recall of 0.97, and an F1-score of 0.98 across 10,746 instances, indicating highly accurate predictions with few false positives. Class 1, comprising 3,460 instances, demonstrated a precision of 0.93, a recall of 0.99, and an F1-score of 0.96, indicating the model’s effectiveness in identifying the class, despite slightly lower precision. For Class 2, the model achieved a precision of 0.98, a recall of 1.00, and an F1-score of 0.99 across 6,142 samples, demonstrating near-perfect performance. The model’s overall accuracy on the test set was 98%, confirming its general reliability. Additionally, the macro average precision, recall, and F1-score were 0.97, 0.99, and 0.98, respectively. The weighted averages, which account for class imbalance, were consistently 0.98 across all three metrics.

**TABLE 2:** Classification report of deep learning model.

Class	Precision	Recall	F1-score	Support
0	1.00	0.97	0.98	10,746
1	0.93	0.99	0.96	3,460
2	0.98	1.00	0.99	6,142
Accuracy	0.98			
Macro Avg	0.97	0.99	0.98	20,348
Weighted Avg	0.98	0.98	0.98	20,348

**FIGURE 4:**
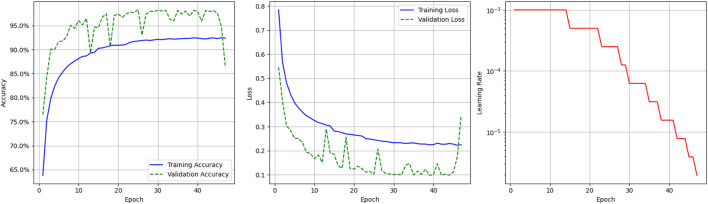
Graphical visualization of deep learning model.

Figure 4 presents the graphical visualization of the deep learning model. It includes three graphs; the first one is the model accuracy graph. The blue line represents the model’s training accuracy, and the dotted green line shows its validation accuracy. The x-axis represents the epoch value, which ranges from 0 to 40, and the y-axis represents accuracy, ranging from 65.0% to 95%. The training accuracy starts at a 0.65% value at the $0_{th}$ epoch and increases to 0.93% at the $40_{th}$ epoch. The validation accuracy starts at 0.76% at the $0^{th}$ epoch. After some fluctuations in increases and decreases, validation accuracy stops at the $40_{th}$ epoch at 87%. The second graph shows the model’s loss (training and validation). The y-axis shows loss values starting from 0.1 to 0.8. The model’s training loss begins at 0.79 in the $0_{th}$ epoch and decreases to 0.21 in the $40_{th}$ epoch. The model’s validation loss starts at 0.55 in the $0_{th}$ epoch and, after some fluctuations, stabilizes at 0.34 in the $40_{th}$ epoch.

The third graph shows the Learning Rate Over Epochs, illustrating the dynamic adjustment of the learning rate throughout the deep learning model’s training. It is defined by the red solid, and the y-axis shows the learning rate range from $10^{-5}$ to $10^{-3}$ . Initially, the learning rate is set at $10^{-3}$ and remains constant for the early epochs, allowing the model to learn rapidly in the initial stages. As training progresses and the validation loss plateaus, the learning rate is reduced in steps. This behavior results from the ReduceLROnPlateau callback, which monitors the validation loss and reduces the learning rate by a factor of 0.5 when no improvement is observed for a predefined number of epochs. This adaptive scheduling continues, with the learning rate progressively decreasing to values as low as $10^{-6}$ , enabling the model to make more refined weight updates in the later stages of training.

**FIGURE 5:**
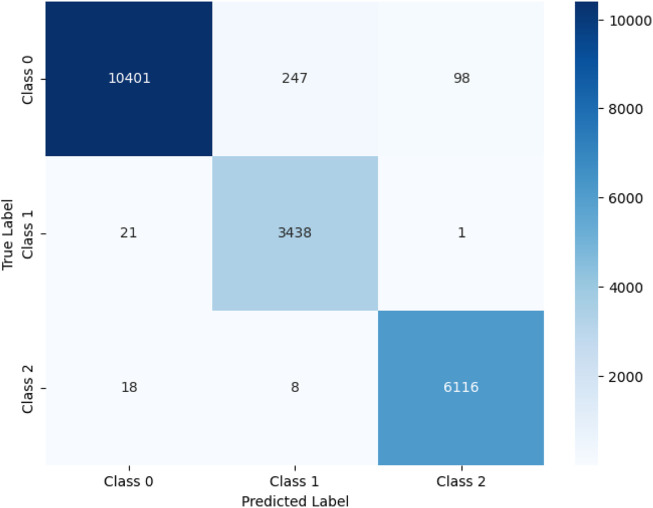
Confusion matrix of 1D-CNN model.

Figure 5 presents a confusion matrix that visually shows the classification performance of the deep learning model across three classes (Class 0, Class 1, and Class 2). Each cell in the matrix indicates the number of model predictions that match the actual labels. This graph reveals that the model exhibits high classification accuracy across all classes. For Class 0, out of 10,746 actual instances, the model correctly predicted 10,401, misclassified 247 as Class 1 and 98 as Class 2. For Class 1, 3,438 out of 3,460 instances were correctly classified, with only 21 instances misclassified as Class 0 and 1 instance misclassified as Class 2. Similarly, for Class 2, the model correctly identified 6,116 out of 6,142 instances, misclassifying 18 as Class 0 and 8 as Class 1.

### 4.2 Results of transfer learning model (ScientiSST MOVE dataset)

Table 3 presents the performance evaluation of the transfer-learning-based deep learning model across 16 classes. The table includes key performance metrics, such as precision, recall, F1-score, and support (i.e., the number of instances) for each class. The model demonstrates strong performance for several classes, particularly Class 0 (run), where precision values are close to 1.00 and recall values are close to 0.9, indicating highly accurate and consistent predictions. However, the performance varies across other classes. For instance, Class 1, Class 2 and Class 3 exhibit relatively lower precision (0.39, 0.40 and 0.14, respectively), suggesting a higher rate of false positives for these categories. Despite this, recall for these classes remains relatively high, particularly for Class 3 (0.94), indicating that most actual instances were correctly identified. The model’s average accuracy across the entire test dataset (13,936 samples) is 82%, with a macro average F1-score of 0.54 and a weighted average F1-score of 0.85.

**TABLE 3:** Classification report of transfer learning model.

Class	Precision	Recall	F1-score	Support
Run	0.99	0.84	0.91	12137
walk_after	0.39	0.60	0.47	969
baseline	0.40	0.71	0.52	699
walk_before_downstairs	0.14	0.94	0.25	131
Accuracy	0.82			
Macro Avg	0.48	0.77	0.54	13936
Weighted Avg	0.91	0.82	0.85	13936

**FIGURE 6:**
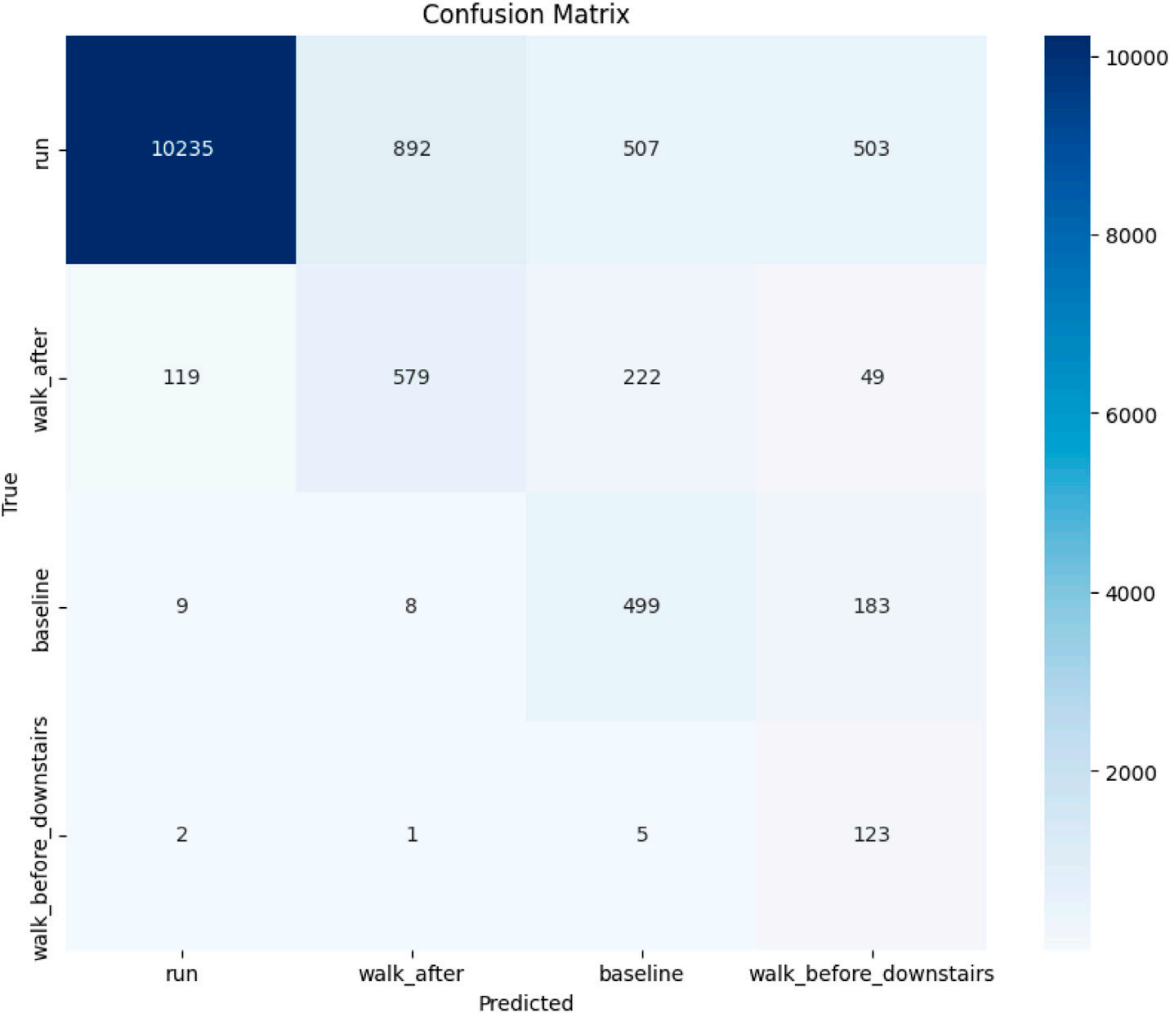
Confusion matrix graph of transfer learning model.

Figure 6 presents the confusion matrix heatmap that provides a comprehensive visual representation of the performance of the transfer learning model across 4 distinct classes (labeled by class name). In this matrix, the true labels are plotted along the vertical axis, and the predicted labels along the horizontal axis. The intensity of the blue color in each cell reflects the number of instances; darker shades indicate a higher count of predictions. Ideally, accurate predictions are located along the diagonal of the matrix, where the predicted labels match the true labels. The model shows strong classification performance for several classes. Notably, Class run stands out with 10235 correct predictions, indicating excellent accuracy and minimal confusion with other classes. Similarly, Classes walk_after, baseline, and walk_before_downstairs also exhibit high accuracy, with most of their predictions concentrated along the diagonal.

#### 4.2.1 Grad-CAM with single channel

**FIGURE 7:**
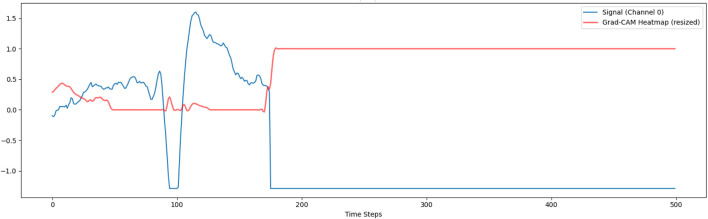
GradCam visualization on single channel.

To improve the interpretability of the deep learning model, we employed Gradient-weighted Class Activation Mapping (Grad-CAM), a widely used XAI technique. Initially, Grad-CAM was applied to a single input channel to visualize the temporal regions contributing most to the model’s decision. Specifically, gradients of the predicted class were backpropagated to the final 1D convolutional layer, generating a class-discriminative heatmap. One representative test sample was selected, and the resulting activation map was upsampled and overlaid on the original signal, as shown in Figure 7. This figure suggests that this method is not providing meaningful localization. The flat response is likely due to a mismatch between the features the model learned on the stress dataset and the patterns in the activity recognition dataset. As a result, the model does not focus on specific temporal regions, which limits the interpretability of the explanation.

#### 4.2.2 Grad-CAM with multiple channels

**FIGURE 8:**
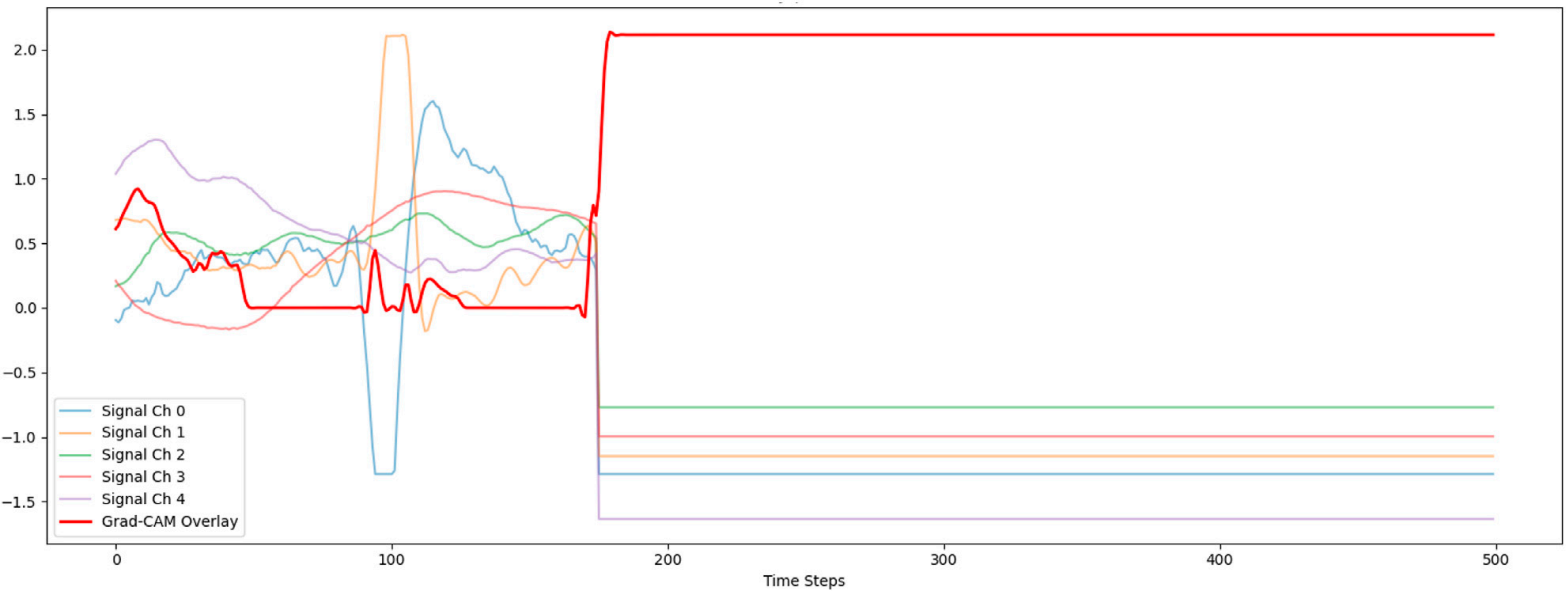
GradCam visualization on multiple channel.

To gain a broader understanding of the model’s attention across all sensor modalities, we extended Grad-CAM to the multichannel setting. This approach computed weighted gradients from the final convolutional layer across all input channels. The aggregated heatmap was then aligned with the time axis of the input data and normalized across channels. Figure 8 shows the overlay of the Grad-CAM heatmap on multichannel signal traces, highlighting both temporal and sensor-specific relevance. This comprehensive interpretation revealed which modalities and time windows carried the most predictive information, thus offering actionable insights into the model’s reasoning and enhancing its transparency in decision-making. Similar to the first figure, the Grad-CAM response becomes flat and fails to align with signal variations. The constant activation indicates that the model is relying on broad, global patterns rather than channel- or time-specific features. This again reflects that the model was pre-trained on stress-related signals and then transferred to activity recognition, potentially leading to misaligned learned features. Consequently, the explanations produced by Grad-CAM appear uninformative and do not clearly indicate which parts of the signals influence the prediction.

### 4.3 Results of temporal conformer model (DREAMER dataset)

Table 4 summarizes the performance of the proposed Temporal Conformer model across multiple evaluation metrics. The model achieved a Mean Absolute Error (MAE) of 0.2400 and a Root mean square error (RMSE) of 0.3330, indicating a low average prediction error and good overall accuracy. The *R* ^2^ score of 0.7826 further demonstrates that the model explains a significant portion of the variance in the target variables, reflecting strong predictive capability. In terms of classification reliability, the rounded accuracy across all output dimensions was 87.59%, i.e., the percentage of samples for which all predicted values matched the true values exactly after rounding. When evaluating each output dimension individually, the model achieved 85.06% accuracy for dimension 1, 91.09% for dimension 2, and 86.63% for dimension 3. These results suggest that the model performs consistently well across all target variables, with particularly high precision in the second dimension.

**TABLE 4:** Evaluation metrics summary for temporal conformer model.

Metric	Value
Mean Absolute Error (MAE)	0.2400
Root mean square error (RMSE)	0.3330
*R* ^2^ Score	0.7826
Rounded Accuracy (All Dimensions)	87.59%
Accuracy: Dimension 1	85.06%
Accuracy: Dimension 2	91.09%
Accuracy: Dimension 3	86.63%

**FIGURE 9:**
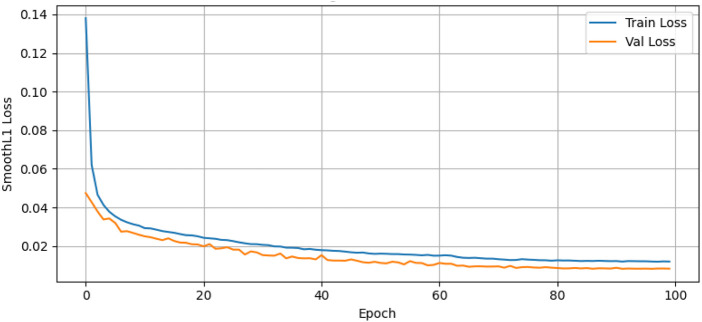
Training and validation loss of temporal conformer model.

Figure 9 presents the training and validation loss of the Temporal Conformer model over 100 training epochs using the Smooth L1 Loss (Huber Loss) as the evaluation metric. The blue line represents the training loss, while the orange line shows the validation loss. The training loss starts at a value of about 0.14 at the $0_{th}$ epoch and decreases to 0.02 at the $40_{th}$ epoch. The validation loss starts at 0.05 in the $0_{th}$ epoch and decreases to 0.01 in the $40_{th}$ epoch.

**FIGURE 10:**
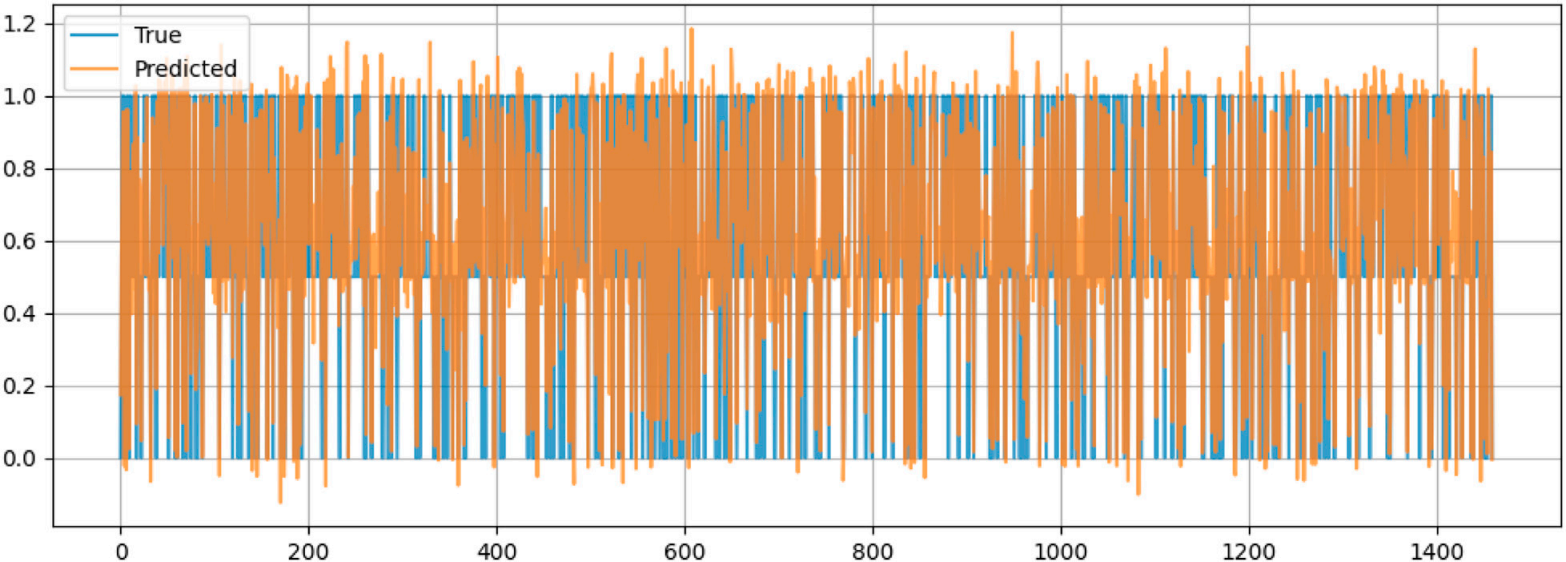
Valence prediction.

Figure 10 compares the actual values (True) and the predicted values (Predicted) of the dataset for valence, which is likely a measure related to emotional tone or sentiment. The x-axis represents a sequence of data points, possibly corresponding to time or to a specific sample index. At the same time, the y-axis ranges from 0 to 1.2, indicating the scale of the valence scores. The blue line represents the true values, and the orange line shows the predicted values. The two sets of values exhibit significant overlap across much of the data range, suggesting that the model’s predictions closely match the actual values. Then there are noticeable discrepancies, particularly where the predicted values (orange) frequently extend both above and below the true values (blue), indicating fluctuating performance by the predictive model. It also reveals extreme spikes and a few instances where the predicted values markedly deviate from the true values, indicating periods of increased prediction error.

Table 5 diagnostic output examples (Rule-Based and Clustered Emotional States). The clustering results provide an alternative categorization of emotional states that does not rely on predefined rules, thereby capturing latent patterns in the data.

**TABLE 5:** Diagnostic output examples (rule-based and clustered emotional States).

Sample	Arousal	Valence	Dominance	Rule-based state	Cluster	Fatigue index	Motion quality
0	0.54	0.56	0.38	Neutral	2	0.46	0.21
1	0.34	0.02	0.33	Passive	3	0.66	0.01
2	0.33	0.06	0.62	Neutral	3	0.67	0.04
3	0.66	0.63	0.37	Neutral	0	0.34	0.23
4	0.31	0.54	0.99	Neutral	1	0.69	0.54
5	0.36	0.57	0.38	Passive	2	0.64	0.21
6	0.09	0.93	0.38	Relaxed	1	0.91	0.35
7	0.34	0.08	0.66	Neutral	3	0.66	0.05
8	0.37	0.47	0.04	Passive	2	0.63	0.02
9	0.63	0.09	0.04	Stressed	0	0.37	0.00

**FIGURE 11:**
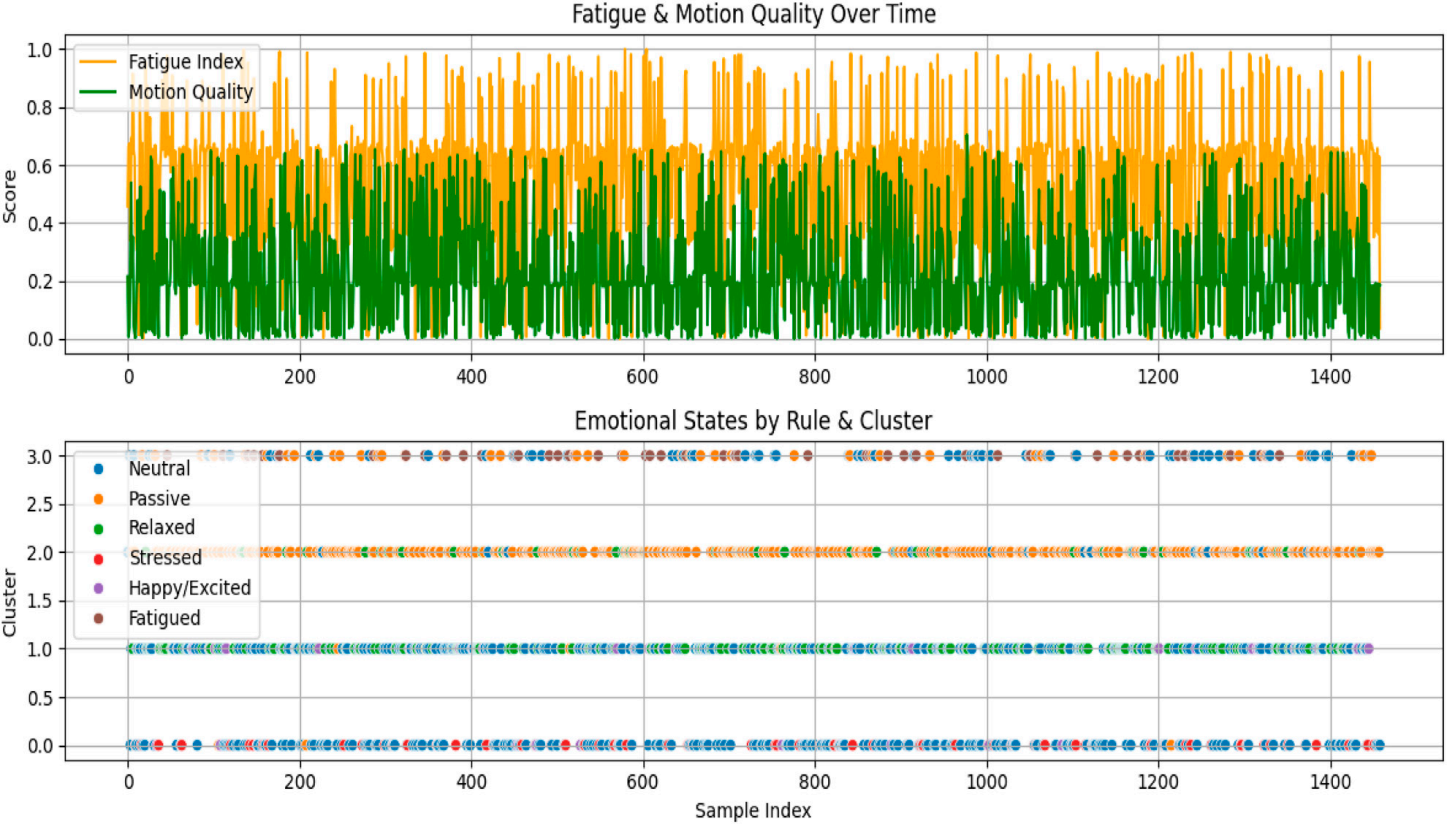
Graphical visualization of emotional state diagnostics.

Figure 11 illustrates two key diagnostic trends in a single view: the top plot shows how Fatigue Index and Motion Quality Score fluctuate over time, reflecting variations in user engagement and tiredness; the bottom scatter plot compares rule-based emotional states with cluster-based groupings, revealing the consistency and divergence between the two classification approaches across samples.

### 4.4 Comparison with existing work

Table 6 presents a comparative analysis of the proposed framework against existing state-of-the-art approaches using various emotion and stress recognition datasets. It shows that existing models such as VGG-16 and RNNs achieved 91% and 93% accuracy, respectively, on the WESAD dataset. Other studies on the DREAMER dataset reported ranges of 80%–90% using ensemble models, while some methods showed very low performance, such as 28%–44%. In contrast, our proposed framework achieved 98% accuracy with the deep learning classifier, 82% accuracy using transfer learning on the complex ScientISST-MOVE dataset, and 87.59% rounded accuracy using the temporal conformer on the DREAMER dataset. These results not only exceed prior benchmarks but also demonstrate superior generalization on more challenging, realistic datasets. Our models combine high accuracy with interpretability, robustness, and cross-domain adaptability, making them more suitable for real-time mental health monitoring applications.

**TABLE 6:** Result comparison with existing approaches.

References	Model	Dataset	Accuracy
Bansal et al. (2025)	VGG-16 with transfer learning	WESAD Dataset	91%
Abdelfattah et al. (2025)	RNN	WESAD Dataset	93%
Ye et al. (2024)	Ensemble (SVM + RF), MLR, RF	DREAMER Dataset	80%–90%
Proposed Framework	Deep Learning Classifier (1D-CNN), + Transfer Learning) + Temporal Conformer (CNN + Transformer)	WESAD Dataset (Stress), ScientISST-MOVE Dataset (Activity), DREAMER Dataset (Emotion)	98%, 82%, 87.59%

### 4.5 Discussion and limitation

This study has several implications that are both significant in terms of research and in the practical fields. The suggested cross-domain deep learning architecture demonstrates that physiological measurements from diverse datasets can be effectively used to identify stress, distinguish activity conditions, and estimate emotional dimensions using a standard architecture. This inter-dataset flexibility highlights the promise of transfer learning and time-based attention systems in overcoming dataset dependency–a long-standing weakness in affective computing. Given its high performance on the WESAD, ScientISST-MOVE, and DREAMER datasets, the framework provides a scalable foundation for multimodal affect recognition systems that generalize across a single data source. Medically, this work is one of the resources that can be used to develop smart, non-invasive systems to track emotional and stress reactions continuously. These may be incorporated into wearable or mobile health technologies to detect stress-related disorders at earlier stages, monitor mental health in the workplace, and provide customized interventions for wellbeing. Combining explainable AI, such as Grad-CAM, Integrated Gradients, and attention visualization, can further improve interpretability and build user trust, which is important for clinical and consumer acceptance of AI-based physiological monitoring.

On a larger scale, the research provides insight into the significance of transfer learning and temporal modeling in the creation of emotionally intelligent systems. The provided framework could serve as a model for future studies on multimodal affect analysis, enabling reproducible, extendable research using a wide range of physiological data. Furthermore, the contributions to methodology, particularly the application of a temporal conformer to continuous emotion prediction, offer opportunities to incorporate affective state prediction into adaptive interfaces, human-computer interaction, and stress management systems. Comprehensively, the implications extend beyond emotion recognition to the architecture of trustful, cross-domain, and context-aware health monitoring systems that can be deployed in the real world. Although the proposed framework achieved excellent, robust results across various datasets, several factors may affect its generalizability. The datasets used, although rather varied, are not very large and were gathered in controlled conditions, which may not be very representative of the variability in physiological or emotional reactions in real-world settings. Moreover, the interpretability of the models is not the final research problem, because visualization methods, such as Grad-CAM, provide only a partial understanding of decision patterns. Finally, the present implementation was optimized for experimental testing and not for resource-constrained systems; consequently, future modifications to wearable or real-time systems may require additional optimization.

## 5 Future work and limitations

The suggested framework is shown to be performing well with high adaptability to various physiological collections, which indicates a possibility of scalable and effective affective-state recognition. However, several avenues could be pursued to extend and enhance this research. Future research can also investigate a broader range of data and sensor sources to confirm the framework across different demographic, environmental, and hardware factors. This would enable a more detailed consideration of the generalizability without sacrificing the methodological premise created in this work. The transfer learning approach that is used in this case has demonstrated significant cross-domain adaptation potential. Following this achievement, a follow-up study can explore domain generalization or self-supervised pretraining methods to enhance further the ability to adapt to heterogeneous or unseen data distributions. The strategies would be an addition to the existing methodology and not a replacement. Interpretability is another field of interest in affective computing that is continuously growing. Although Grad-CAM helped visualize explanations, other explainability methods, such as SHAP or Integrated Gradients, may be included in future studies to provide a more physiological explanation of what the learned representations mean. This would deepen the transparency without changing the fundamentals made in this paper.

Even though this piece of work employed well-controlled benchmark data, including WESAD and DREAMER, to guarantee reproducibility and comparability, the same structure may be successfully applied to real-world or longitudinal data collected with wearable sensors. It would be valuable to evaluate the model’s real-time and environmental performance in naturalistic environments using the current architecture. Lastly, although computational requirements in this study were satisfactorily handled, the architecture can be further streamlined for edge or mobile deployment in future applications. Small systems such as model compression, effective feature extraction, or hardware-sensitive tuning would be helpful for integration with wearable or continuous monitoring systems. The suggested framework, in general, provides a solid basis for physiological emotion and stress detection. The directions listed here are its logical extensions, developed from its proven usefulness, opening the path to a wider scope of application, greater interpretability, and practical deployment.

## 6 Conclusion

This study presents a comprehensive dual-approach framework for emotion and stress recognition using physiological signals, demonstrating significant advancements enabled by XAI integration. The first proposed approach introduces a framework that utilizes deep learning-based transfer learning by pretraining a 1D-CNN on the WESAD dataset and fine-tuning it on the ScientISST-MOVE dataset, improving cross-domain adaptability for stress and activity classification. The second approach uses a Temporal Conformer architecture on the DREAMER dataset to continuously predict emotional dimensions, including VAD, thereby capturing the temporal dynamics of affective states. Both pipelines incorporate advanced preprocessing, feature extraction, and techniques for handling class imbalance to enhance model robustness and generalization. Achieving classification accuracy rates exceeding 98%, the proposed framework holds strong potential for real-time, interpretable, and personalized mental health monitoring. Specifically, the deep learning classifier on biosignal data achieved 98% accuracy and a macro F1-score of 0.98 across three classes. The transfer learning model, fine-tuned on the ScientISST-MOVE dataset, textcolorblueattained 82% accuracy across classes with strong performance across major categories. However, explanations produced by Grad-CAM appear uninformative and do not clearly indicate which parts of the signals influence the prediction. Meanwhile, the temporal conformer model applied to the DREAMER dataset achieved an *R* ^2^ score of 0.78 and a rounded accuracy of 87.59% across valence, arousal, and dominance detection. Future research will aim to expand the framework’s applicability across diverse biosignal domains, incorporate real-world deployment scenarios, and explore scalability for broader applications in affective computing.

## Data Availability

The original contributions presented in the study are included in the article/supplementary material, further inquiries can be directed to the corresponding authors.

## Footnotes

1
https://www.kaggle.com/datasets/phhasian0710/dreamer

2
https://archive.ics.uci.edu/dataset/465/wesad+wearable+stress+and+affect+detection
